# Second-line oxaliplatin reintroduction or paclitaxel-ramucirumab in metastatic gastroesophageal cancer: a population-based study

**DOI:** 10.1177/17588359261442616

**Published:** 2026-06-03

**Authors:** Sebastiaan N. Siegerink, Steven C. Kuijper, Tom van den Ende, Rob H. A. Verhoeven, Sjoerd G. Elias, Nadia Haj Mohammad, Marije Slingerland, Sarah Derks, Hanneke W. M. van Laarhoven

**Affiliations:** Department of Medical Oncology, Amsterdam UMC, Office 8D-XXX, PO Box 22660, Amsterdam 1100DD, The Netherlands; Cancer Center Amsterdam, Cancer Treatment and Quality of Life, Amsterdam, The Netherlands; Department of Medical Oncology, Amsterdam UMC Location University of Amsterdam, Amsterdam, The Netherlands; Cancer Center Amsterdam, Cancer Treatment and Quality of Life, Amsterdam, The Netherlands; Department of Medical Oncology, Amsterdam UMC Location University of Amsterdam, Amsterdam, The Netherlands; Cancer Center Amsterdam, Cancer Treatment and Quality of Life, Amsterdam, The Netherlands; Department of Medical Oncology, Amsterdam UMC Location University of Amsterdam, Amsterdam, The Netherlands; Cancer Center Amsterdam, Cancer Treatment and Quality of Life, Amsterdam, The Netherlands; Department of Research and Development, Netherlands Comprehensive Cancer Organisation (IKNL), Utrecht, The Netherlands; Department of Epidemiology, Julius Center for Health Sciences and Primary Care, University Medical Center Utrecht, Utrecht University, Utrecht, The Netherlands; Department of Medical Oncology, University Medical Center Utrecht, Utrecht University, Utrecht, The Netherlands; Department of Medical Oncology, Leiden University Medical Center, Leiden, The Netherlands; Department of Medical Oncology, Amsterdam UMC location University of Amsterdam, Amsterdam, The Netherlands; Cancer Center Amsterdam, Cancer Treatment and Quality of Life, Amsterdam, The Netherlands; Oncode Institute, Amsterdam, The Netherlands; Department of Medical Oncology, Amsterdam UMC, Office 8D-XXX, PO Box 22660, Amsterdam 1100DD, The Netherlands; Cancer Center Amsterdam, Cancer Treatment and Quality of Life, Amsterdam, The Netherlands

**Keywords:** beyond first-line treatment, esophagogastric cancer, oxaliplatin reintroduction, paclitaxel-ramucirumab, target trial emulation

## Abstract

**Background:**

Reintroduction of oxaliplatin after first-line treatment might preserve treatment lines and could improve overall survival.

**Objectives:**

We investigate whether oxaliplatin reintroduction (OXR) or paclitaxel-ramucirumab (PR) is preferred in terms of overall survival for patients with gastroesophageal adenocarcinoma who have disease progression on first-line treatment.

**Design:**

We performed a target trial emulation with a propensity-scored inverse probability weighting approach to resemble a randomized controlled trial.

**Methods:**

Observational data was retrieved from the Netherlands Cancer Registry between 2015 and 2018, with a maximum follow-up until 2022. Patients were included if they received a fluoropyrimidine with oxaliplatin in first-line and as subsequent treatment OXR or PR. There was at least 6 months between planned discontinuation of oxaliplatin and disease progression.

**Results:**

We included 129 patients in our model and the weighted cohort was well balanced between the two groups. No statistically significant difference in overall survival was observed between treatment groups (HR: 0.76, 95% CI: 0.3–1.5; *p* = 0.36). Median overall survival was 9.2 months (95% CI: 3.7–25.1) for OXR and 7.9 months (95% CI: 2.8–11.5) for PR (*p* = 0.38). Exploratory subgroup analysis did not reach statistical significance. Patients in the OXR-group more often received one or more additional treatment lines, including second-line PR, than patients in the PR group, 47.7% versus 23.3%, respectively.

**Conclusion:**

OXR following prolonged response to first-line treatment was associated with similar overall survival compared with PR. In the absence of a demonstrated survival benefit for either treatment, these results propose oxaliplatin reintroduction as a potential option in selected patients.

## Background

The global impact of gastroesophageal cancer is significant with a worldwide incidence of 1.7 million cases per year, and 1.3 million deaths.^1,2^ Future projections predict the incidence to increase to nearly 3 million people by 2040.^1,2^ The prognosis is poor with a 1-year overall survival (OS) of around 20% in the metastatic setting for gastroesophageal cancer.^3^ In first-line, the majority of patients receives doublet chemotherapy with a fluoropyrimidine and a platinum compound, that is, 5-fluorouracil (5-FU) or capecitabine and oxaliplatin or cisplatin, respectively, combined with targeted agents and/or checkpoint inhibitors depending on the tumor biomarker profile.^4–7^ A common side effect of oxaliplatin is polyneuropathy, which can be irreversible without intervention. Polyneuropathy is detrimental for quality of life, and may be the reason for dose reductions or discontinuation of treatment altogether.^8–10^ Moreover, due to the clear dose response relationship, platinum compounds are often withheld after 4–6 months to prevent cumulative (neuro)toxicity. The fluoropyridine and/or the targeted agent or checkpoint inhibitor may be continued as maintenance treatment.^11,12^

In colorectal cancer, it has been postulated that, after disease progression during maintenance treatment, reintroduction of oxaliplatin could be beneficial to preserve treatment options by postponing second-line treatment and, subsequently, improve OS. In a previous systematic review oxaliplatin reintroduction in metastatic colon cancer was defined as “retreatment with oxaliplatin-based regimens from which patients had previously had disease control and to which the tumor has not yet proved to be resistant.”^12^ In most of the included studies, patients were required to have an “oxaliplatin-free interval” of at least 6 months.^12^ Previously, a randomized controlled trial, OPTIMOX1, demonstrated that reintroduction of oxaliplatin after planned discontinuation after six cycles in patients with prolonged response or stable disease was associated with lower toxicity and reached similar OS when compared to continuous chemotherapy followed by second-line treatment upon progression, with a median OS of 20.7 and 21.4 months (*p* = 0.75).^13^

In metastatic gastroesophageal cancer, reintroduction of oxaliplatin has not been extensively researched. The retrospective cohort study AGEO, compared maintenance fluoropyrimidine with trastuzumab to trastuzumab alone after induction with platinum, fluoropyrimidine, and trastuzumab in HER2^+^ patients.^14^ Upon progression, patients in this cohort received reintroduction of oxaliplatin (*N* = 26) or a variety of second-line treatment combinations (*N* = 86), including paclitaxel-ramucirumab (*N* = 24). Median OS in patients receiving oxaliplatin reintroduction was better than other second-line treatment regimens, 13.8 months versus 9.0 months, respectively (*p* = 0.007).^14^ However, in this retrospective cohort study, the decision to reintroduce oxaliplatin may have been influenced by its association with more favorable prognostic indicators such as good response to first-line treatment and a better performance score. Moreover, these results only pertain to a HER2^+^ population, where reintroduction might be preferred as a means to continue HER2-targeting treatment. Furthermore, the study did not directly compare oxaliplatin reintroduction to standard of care second-line treatment, paclitaxel-ramucirumab.^15^

As illustrated above, evidence is scarce, and there are no randomized controlled trials comparing reintroduction of oxaliplatin to second-line treatment options. Therefore, the aim of this study is to investigate whether reintroduction of oxaliplatin or paclitaxel-ramucirumab is preferred in terms of OS for patients with gastroesophageal adenocarcinoma who have disease progression on first-line treatment with a fluoropyrimidine and oxaliplatin and a prolonged response of at least 6 months after (planned) discontinuation of oxaliplatin. This will be done by emulating a target trial with the use of propensity-scored inverse probability weighting (PS-IPW) of a retrospective cohort retrieved from the Netherlands Cancer Registry (NCR). PS-IPW is a useful tool to account for measured confounders when comparing effectiveness of treatments from observational data and it is increasingly used in the field of medicine.^16^ Hernán and Robins approach for a target trial emulation offers a framework that—combined with the appropriate statistical methods—approaches a randomized controlled trial, when said trial is not feasible or timely.^17^

## Methods

We performed a target trial emulation to resemble a randomized controlled trial as closely as possible with our observational data. Therefore, we included patients with adenocarcinomas in the esophagus, GE-junction or stomach (gastroesophageal carcinoma or GEC), treated with capecitabine or 5-FU and oxaliplatin as first-line treatment (CAPOX/FOLFOX, respectively), who then discontinued oxaliplatin for at least 6 months, while maintaining stable disease, partial response, or complete response. Maintenance treatment with capecitabine or 5FU and trastuzumab (if applicable) would be allowed. Patients would be excluded if they received any other treatment. Upon disease progression, patients would be randomized for treatment with reintroduction of oxaliplatin or paclitaxel-ramucirumab, the standard of care second-line treatment in the Netherlands. The starting time of a randomized controlled trial would be time of randomization; however, due to the retrospective nature of this cohort, the chosen time zero is start of second-line treatment. In the setting of second-line treatment, usually there is limited time between randomization and start of treatment. For example, in the RAINBOW trial, which analyzed the addition of ramucirumab to paclitaxel, treatment had to initiate within 7 days of randomization.^18^ Therefore, we believe that defining time zero as the start of second-line treatment is the most accurate and appropriate approach, and that immortal time bias is unlikely to influence our findings. This target trial framework was based on previous work by Hernan and Robins^17^ and is illustrated in Table 1. The reporting of this study conforms to the EQUATOR STROBE (Strengthening the Reporting of Observational Studies in Epidemiology) and RECORD (REporting of studies Conducted using Observational Routinely collected health Data) checklists for observational studies.^19,20^

**Table 1.** Target trial emulation of our data comparing oxaliplatin reintroduction to paclitaxel-ramucirumab.

Target trial emulation	
Eligibility	*Inclusion criteria*: Patients with metastatic gastroesophageal adenocarcinoma diagnosed between 2015 and 2018 and registered in the Netherlands Cancer Registry were eligible. Patients must have received first-line fluoropyrimidine-based chemotherapy (capecitabine or 5-fluorouracil) in combination with oxaliplatin; targeted therapy may have been added when applicable. Eligible patients subsequently received second-line treatment with either oxaliplatin reintroduction combined with a fluoropyrimidine or paclitaxel-ramucirumab after an oxaliplatin-free interval of at least 6 months without documented disease progression *Exclusion criteria*: Patients were excluded if oxaliplatin was reintroduced within 6 months of discontinuation of first-line oxaliplatin, if they had squamous cell carcinoma histology, if they received a non–oxaliplatin-based first-line regimen, or if they received a second-line regimen other than paclitaxel-ramucirumab or oxaliplatin reintroduction
Treatment	Reintroduction of oxaliplatin together with the fluoropyrimidine and, if applicable, targeted therapy (given in the same way as first-line treatment) Paclitaxel and ramucirumab according to standard protocol
Assignment procedure	Participants were not randomly assigned but obtained from observational real-world data. To control for unobserved confounding, we will include the following predetermined treatment outcome-related variables: age, sex, performance status, HER2 status, lactate dehydrogenase and number of metastatic sites as a proxy for tumor load, primary tumor location (stomach or esophageal), duration of first-line treatment, and neuropathy during first-line treatment—defined by discontinuation of first-line treatment due to neuropathy
Time zero and follow-up	Time zero was defined as the start of second-line treatment. Since there was no randomization, the true time of randomization can only be approximated due to the retrospective nature of this emulation Follow-up ends at death or loss to follow-up. Maximum follow-up time was 8 years. There was no censoring, none of the patients were alive by the time of last follow-up
Outcome	Overall survival
Causal contrast of interest	Intention-to-treat effect

The data were collected from the NCR, a nationwide population-based database that systematically collects data on all cancer diagnoses in the Netherlands. Patients were selected based on their registered tumor code (C15 and C16 for stomach and esophagus) and cT4b and cM1 tumors. The NCR does not routinely register beyond first-line treatments, except for the specific cohort of patients diagnosed with synchronous metastatic disease from 2015 until 2018. At this time, checkpoint inhibition was not yet approved in the Netherlands for GEC, hence no included patients received PD-1 inhibitors as first-line and maintenance treatment. Inclusion and exclusion criteria can be found in Table 1. Records with treatment misclassification were excluded during data cleaning. For example, a fluoropyrimidine switch from capecitabine to 5-FU in first-line was erroneously interpreted as oxaliplatin reintroduction in the NCR and was therefore excluded.

To identify possible confounders, a directed-acyclic graph (DAG) was created with help of medical oncologists (N.H., S.D., M.S.) specialized in GEC from three university medical centers in the Netherlands, (Supplemental Figure 1). Based on the DAG, several prediction models^21–24^ and the SOURCE^25^ beyond first-line survival prediction model for patients with metastatic gastroesophageal adenocarcinoma, which was developed in the Amsterdam UMC and used data from 1067 patients, including the 129 patients in the cohort of this study. Nine variables related to OS were included: age, sex, performance status, HER2 status, lactate dehydrogenase (LDH) and number of metastatic sites as a proxy for tumor load, primary tumor location (stomach or esophageal), duration of first-line treatment, and neuropathy during first-line treatment – defined by discontinuation of first-line treatment due to neuropathy. Other toxicity on first-line treatment was suggested by the panel of medical oncologists, but these were not recorded by the NCR. However, toxicities that affect choice of treatment would at least partially be captured by the performance status, which can serve as a proxy.

To control for the aforementioned possible confounders, we employed PS-IPW. PS-IPW has been used extensively in medical research and has been described as a reliable method to mimic randomized controlled trials.^26^ Using this model—under the assumption of no unmeasured confounding—the results would represent the outcomes of the entire sample had all patients received reintroduction of oxaliplatin, as well as, the outcomes of the entire sample had they all received paclitaxel-ramucirumab.^27^ This estimates the average treatment effect (ATE). We used a multivariable logistic regression model to estimate the propensity of receiving reintroduction of oxaliplatin versus paclitaxel-ramucirumab using the aforementioned covariates. Standardized mean differences (SMDs) were calculated to assess covariate balance between treatment groups, with an SMD ⩽0.10 indicating adequate balance. Interaction terms were sequentially added to the logistic model until all covariates met this threshold. To improve covariate overlap, patients with propensity scores outside the 2.5th and 97.5th percentiles were trimmed. Patients were subsequently weighted based on the propensity score. The median weight was 1.6, and no weight exceeded 7. Furthermore, to assess the weighted model’s ability to adjust for confounding we performed a post-PS-IPW c-index, in which we tested the model’s ability to predict which treatment patients would have received based on the confounders present in the model. A score of 0.5 would indicate that the model cannot predict treatment based on the selected confounders and that the model correctly adjusts for the confounders. Without weights the c-index was 0.71, after weighting it was 0.54.

All analyses were conducted using RStudio version 4.4.3.^28^ Statistical significance was defined as a two-sided *p* value less than 0.05. Missing data were assessed using the MissMech package,^29^ and the null hypothesis of data being missing completely at random was rejected. Variables with an excessive proportion of missing data (e.g., differentiation grade) were excluded from multivariable models. Subsequently, the missing variables LDH (12 missing values) and performance status (19 missing values) were imputed with single imputations with the missForest implementation for R.^30^ This applies random forest imputation with predictive mean matching. Predictor variables were selected based on clinical relevance. Imputed datasets were used for all subsequent analyses. Kaplan–Meier curves were created and assessed using a log rank test. Cox proportional hazards regression was used to estimate hazard ratios (HRs) for the ATE and for predefined subgroups. All variables included in the PS-IPW model were incorporated into the subgroup analyses. Bootstrapping of the Cox models was performed to improve the accuracy and reliability of confidence intervals and *p* values, as recommended when using PS-IPW with sample sizes smaller than 1000.^31^ As a sensitivity analysis, we fitted a multivariable-adjusted Cox proportional hazards model to assess whether conclusions were consistent under conditional effect estimation. The proportional hazards assumption was assessed using Schoenfeld residuals. The subgroup analysis was plotted with the R package Forester.^32^ Here binary variables were created from continuous variables (age, performance status, LDH, and number of metastases) based on their median.

OS was defined as the time from initiation of either oxaliplatin reintroduction or paclitaxel-ramucirumab to death from any cause or emigration as recorded in the Dutch Municipal Personal Records Database, per protocol of the NCR. Subsequent systemic therapies, including oxaliplatin rechallenge in patients treated with second-line paclitaxel-ramucirumab, were considered part of routine clinical care and were not censored. Under this approach, outcomes are evaluated regardless of postbaseline treatment modifications, reflecting real-world clinical practice and aligning with pragmatic trial principles, which is in accordance with European Medicines Agency’s adoption of the International Council for Harmonisation guideline E9.^33^

## Results

A total of 6282 patients diagnosed with cT4b and/or cM1 GEC between 2015 and 2018 were screened for inclusion by the NCR. The NCR selected 358 patients who received CAPOX or FOLFOX as first-line treatment and upon progression received oxaliplatin or paclitaxel-ramucirumab and were sent to us in April 2024. The date of final follow-up for this cohort was 2022. Another 229 patients were excluded because they met the exclusion criteria (Supplemental Figure 2). A total of 129 patients were initially eligible for inclusion in the propensity score inverse probability weighted model (Supplemental Figure 2). After trimming patients with propensity scores outside of the 2.5th and 97.5th percentiles this was reduced to 121. Weighting resulted in a well-balanced pseudopopulation of 241.5 patients, as demonstrated by the SMD <0.1 in Table 2.

**Table 2.** Baseline characteristics table before and after adjustment with propensity-scored inverse probability weighting, frequencies presented as “No. (%)” unless stated otherwise.

Variables	All patients			Patients after PS-IPW		
	Reintroduction oxaliplatin	Paclitaxel-ramucirumab	SMD	Reintroduction oxaliplatin	Paclitaxel-ramucirumab	SMD
All patients	51	70		121.5	120.0	
Age in years (mean, SD)	62.9 (8.0)	63.0 (10.1)	0.018	62.9 (8.1)	62.9 (9.8)	0.002
Sex			0.272			0.071
Female	7 (13.7)	17 (24.3)		20.2 (16.6)	23.2 (19.3)	
Male	44 (86.3)	53 (75.7)		101.3 (83.4)	96.8 (80.7)	
Performance status			0.009			0.023
0	25.0 (49.0)	34.0 (48.6)		57.5 (47.3)	58.2 (48.5)	
1 or higher	26.0 (51.0)	36.0 (51.4)		64.0 (52.7)	61.8 (51.5)	
LDH (mean, SD)	234.1 (130.0)	250.5 (177.5)	0.106	247.9 (167.4)	243.5 (159.0)	0.027
Location of primary tumor			0.362			0.041
Stomach	23 (45.1)	44 (62.9)		69.7 (58.4)	66.4 (55.4)	
Esophagus	28 (54.9)	26 (37.1)		51.8 (42.6)	53.6 (44.6)	
Number of organs with metastases (mean, SD)	1.53 (0.67)	1.60 (0.84)	0.093	1.58 (0.71)	1.55 (0.81)	0.040
Peritoneal metastases			0.178			0.028
Yes	12.0 (23.5)	22.0 (31.4)		31.5 (30.0)	34.5 (28.8)	
No	39 (76.5)	48 (68.6)		85.0 (70.0)	85.5 (71.2)	
HER2			0.065			0.077
Negative	40.0 (78.4)	53.0 (75.7)		88.3 (72.7)	91.2 (76.0)	
Positive	11.0 (21.6)	17.0 (24.3)		33.2 (27.3)	28.8 (25.9)	
First-line PFS, in days (mean, SD)	517.4 (227.3)	428.6 (241.4)	0.379	473.0 (204.0)	460.1 (270.2)	0.054
Neuropathy during first-line treatment			0.168			0.064
Yes	7 (13.7)	14 (20.0)		18.8 (15.5)	21.4 (17.8)	
No	44 (86.3)	56 (80.0)		102.7 (84.5)	98.6 (82.2)	

LDH, lactate dehydrogenase; PFS, progression-free survival; SD, standard deviation; SMD, standardized mean difference.

**Figure 1.**
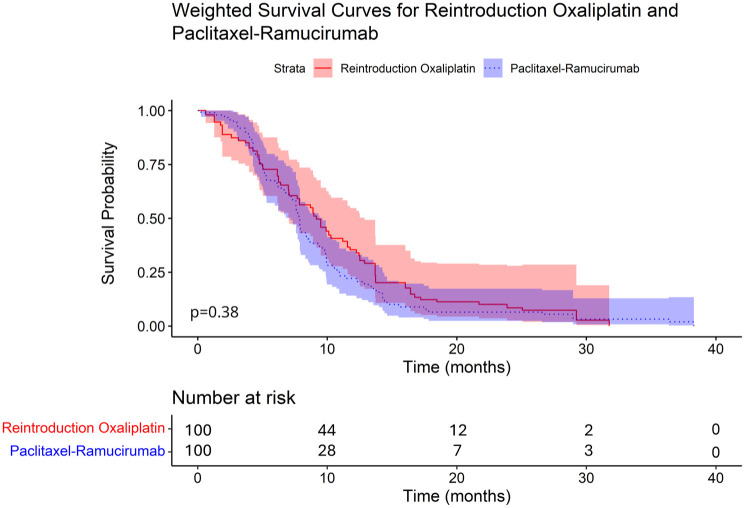
Kaplan–Meier curves of overall survival of patients treated with reintroduction oxaliplatin (continuous line) and paclitaxel-ramucirumab (dotted line) in the PS-IPW population, *p* value calculated with a log rank test. PS-IPW, propensity-scored inverse probability weighting.

There was no statistically significant difference in overall treatment effect between oxaliplatin reintroduction and paclitaxel-ramucirumab, the HR favored oxaliplatin with 0.76, (95% CI: 0.3–1.5, *p* = 0.36). Median OS of the oxaliplatin reintroduction group was 9.2 months (95% CI: 3.7–25.1 months) and of the paclitaxel-ramucirumab group was 7.9 months (95% CI: 2.8–11.5 months, *p* = 0.38; Figure 1). There was no censoring in the Kaplan–Meier curve of Figure 1, since all patients had died by the time of last follow-up. In the sensitivity analysis using a multivariable-adjusted Cox model, the conditional HR was slightly smaller than the marginal PS-IPW estimate, as expected given differences in treatment effect estimates. Importantly, the treatment effect remained directionally consistent.

The mean number of cycles for patients with oxaliplatin reintroduction was 3.8 (standard deviation (SD) 1.9) compared to 5.3 (SD 3.0) cycles of paclitaxel-ramucirumab.

**Figure 2.**
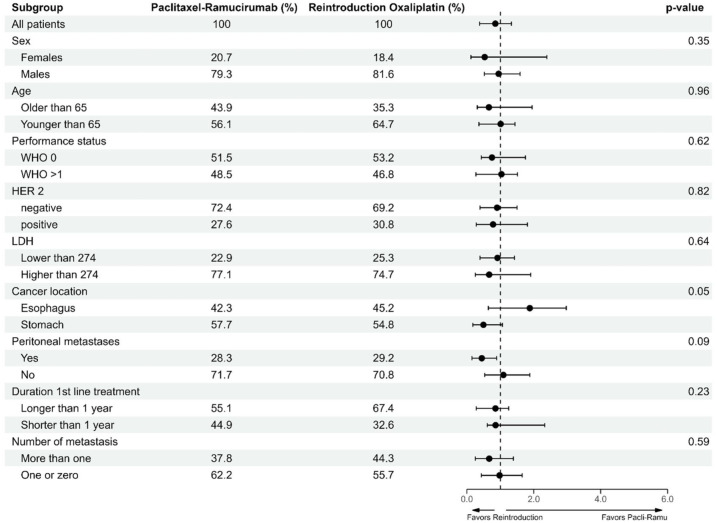
Forest plot showing the subgroup analysis of the treatment effect on overall survival based on baseline characteristics of the PS-IPW population. The dashed line represents a hazard ratio of 1. LDH, lactate dehydrogenase; PS-IPW, propensity-scored inverse probability weighting.

None of the subgroups analyses demonstrated statistically significant differences. Exploratory trends were observed, for example, patients with stomach cancer seemed to have numerically better survival outcomes with oxaliplatin reintroduction (HR: 0.6, 95% CI: 03–1.0), whereas patient with esophageal cancer showed a numerically higher HR when treated with oxaliplatin reintroduction compared to treatment with paclitaxel-ramucirumab (HR: 1.5, 95% CI: 0.8–3.0). Moreover, the presence of neuropathy during first-line treatment was associated with numerically improved outcomes in patients treated with paclitaxel-ramucirumab (HR: 1.7, 95% CI: 0.8–4.7). However, due to the small patient numbers within these subgroups, these pre-planned subgroup analyses lack statistical power and should be interpreted as exploratory and hypothesis generating. This uncertainty is reflected in the wide confidence intervals as shown in the forest plot (Figure 2).

After second progression, patients in the oxaliplatin reintroduction group more often received one or more treatment lines compared to patients in the paclitaxel-ramucirumab group, 48.4% versus 23.7%, respectively (Table 3). The most common treatment after oxaliplatin reintroduction was paclitaxel-ramucirumab, in 15 out of the 22 cases (68%). Subsequent lines in de oxaliplatin reintroduction group were combinations of CAPOX or FOLFOX and paclitaxel-ramucirumab. Subsequent treatment lines in the paclitaxel-ramucirumab group were very heterogenous. The most frequently used regimens were fluoropyrimidine-oxaliplatin combinations, irinotecan, or trifluridine/tipiracil (Supplemental Table 1).

**Table 3.** Overview of survival after start of second-line treatment per treatment group, dependent on how many lines of treatment they received after reintroduction oxaliplatin or after paclitaxel-ramucirumab.

Number of treatment lines beyond second-line	Reintroduction oxaliplatin		Paclitaxel-ramucirumab	
	*N* (%)	Median OS (months)	*N* (%)	Median OS (months)
0	52.6	6.1	77.3	7.3
1	43.3	12.3	15.0	10.0
2	1.3	11.2	6.1	26.6
3	2.7	31.8	1.3	36.4

Patient numbers are affected by PS-IPW and are therefore presented in percentages.

OS, overall survival; PS-IPW, propensity-scored inverse probability weighting.

## Discussion

This is the first cohort study with target trial emulation in gastroesophageal cancer investigating oxaliplatin reintroduction compared to second-line treatment with paclitaxel and ramucirumab. Oxaliplatin reintroduction was associated with similar OS compared with paclitaxel-ramucirumab. However, due to the limited power, it is not possible to definitively distinguish between a moderate survival advantage, no effect or a survival disadvantage.

The hypothesized benefit from reintroduction of oxaliplatin is due to the potential of preserving subsequent treatment lines. In the aforementioned AGEO study by Bergen et al.,^14^ the secondary objective was to compare reintroduction of first-line treatment with second-line treatment. In this population of HER2^+^ patients, a markedly improved OS was found for patients receiving reintroduction of first-line treatment compared with second-line. Interestingly, the progression-free survival (PFS) of the group undergoing reintroduction of oxaliplatin was similar to the PFS in the group with paclitaxel and ramucirumab. This suggests that any observed survival benefit may indeed be caused by the increased number of treatment lines administered beyond reintroduction, provided that neurotoxicity does not prevent further treatment. Thus, rather than describing oxaliplatin reintroduction as a more beneficial treatment than paclitaxel-ramucirumab, it may be more helpful to regard it as a continuation of first-line treatment. This partially parallels the treatment strategy in metastatic colorectal cancer, where oxaliplatin reintroduction strategies were developed primarily within stop-and-go first-line approaches and have not been formally compared with standard second-line regimens in randomized trials.^34^ Due to considerable toxicity and subsequent poorer survival associated with continuous oxaliplatin exposure, intermittent oxaliplatin administration, with maintenance fluoropyrimidines and, when feasible, maintenance anti-Vascular Endothelial Growth Factor (VEGF) or anti-Epidermal Growth Factor Receptor (EGFR) targeted therapies, became the standard of care in metastatic colorectal cancer.^35^ While these strategies were developed in the context of planned treatment de-escalation, in a different organ, with a different biological background, these experiences provide a useful conceptual framework in which we might see reintroduction of oxaliplatin, with some important caveats.

First, it is important to note that this is a selective cohort of patients. In daily clinical practice, in many western countries only a minority of patients proceed beyond first- and second-line therapies, despite the introduction of third-line treatment options such as trifluridine-tipiracil and trastuzumab-deruxtucan.^15^ Patients were only included if they did not progress in the 6 months after the last cycle of oxaliplatin, in other words a PFS of at least 10.5 months. The selection that was made as described above is associated with an exceptional response to first-line treatment and thus, presumably, an exceptional response to oxaliplatin. Total median OS, including first-line treatment was between 18 and 20 months, which is much higher than the approximate 8 months in the general population of metastatic GEC patients receiving systemic treatment.^3^ This prolonged PFS is likely attributable to their tumor biology and might be the reason why these patients benefit from oxaliplatin reintroduction, as they are likely a select group that maintains platinum sensitive, where other patients become platinum resistant. Platinum resistance is a common problem among different types of cancer^36–38^ and it relies on DNA repair mechanisms such as nucleotide excision repair and mismatch repair.^39^ In ovarian cancer, BRCA 1/2 mutations are strongly associated with platinum sensitivity and in bladder cancer ERCC2 mutations have been linked to increased sensitivity to platinum-containing chemotherapy.^40^ In gastroesophageal cancer, biomarker-driven prediction of platinum sensitivity remains less well established. For example, ERCC1 (excision repair cross-complementing group 1), a key mediator of nucleotide excision repair, has been associated with resistance to platinum agents, with studies suggesting that low or absent ERCC1 expression is associated with improved response to oxaliplatin-containing regimes.^38^ However, these findings have not reached the clinic. Moreover, in bladder cancer and ovarian cancer, a more pragmatic approach has been chosen. Duration of response to prior platinum-containing treatment is used as a surrogate marker of platinum sensitivity.^41,42^ Often a platinum-free interval of 6–12 months is used to distinguish the range between partially sensitive and fully sensitive disease.^43^ Unfortunately, due to the retrospective and epidemiological nature of this study, we have no specific data on tumor biology; however, it is expected that this select patient population shares a favorable, platinum-resistant phenotype.

While this is a selective population, now that immune checkpoint inhibition is also available for patients in first-line, more patients will exceed 6 months PFS after the last dose of oxaliplatin and will thus be eligible for oxaliplatin reintroduction.^44^ This introduces the second caveat, which is that this study is based on a cohort selected between 2015 and 2018 and therefore predates the current therapeutic landscape of metastatic gastroesophageal cancer.^6,7^ Including the routine use of immune checkpoint inhibitors in the first-line setting and the implementation of biomarker-drive treatment strategies based on microsatellite instability-high status or a combined positive score, which determines eligibility for immune checkpoint inhibition or Claudin 18.2 expression for targeted treatment. Consequently, treatment outcomes observed in this analysis may not be fully generalizable to contemporary practice. The introduction of immunotherapy or targeted therapy may influence subsequent chemotherapy sensitivity, including the efficacy of oxaliplatin reintroduction. Platinum-based treatments may synergize with immunotherapy and targeted therapy but may also reduce the effectiveness of later platinum reintroduction by immune-driven clonal selection and acquired resistance mechanisms.^45^ At present, clinical data on oxaliplatin reintroduction following first-line chemotherapy with immunotherapy or targeted therapy in gastroesophageal cancer are limited. Nevertheless, this study did include patients receiving HER2-targeted treatment, which did not appear to affect the efficacy of oxaliplatin reintroduction, but that is based on the exploratory analysis of the HER2 positive subgroup. In colorectal cancer, anti-VEGF and anti-EGFR targeting therapies have seamlessly entered the guidelines while maintaining the oxaliplatin reintroduction strategies.^35^ Importantly, paclitaxel-ramucirumab remains the standard second-line regimen for many patients, and thus far novel therapies are restricted to molecularly defined subgroups or later-line settings.^6,7^ Among those patients with actionable alterations, access to targeted or immunotherapeutic options may be limited by eligibility, toxicity, or logistical constraints. These considerations suggest that oxaliplatin reintroduction may remain clinically relevant in selected patients when individualized decisions are required.

Third, in the absence of demonstrable survival benefit, recommending oxaliplatin reintroduction necessitates clear data on neurotoxicity-related harm and patient-reported tolerability. Oxaliplatin-induced neuropathy is a well-recognized dose-limiting toxicity of oxaliplatin-based chemotherapy and can lead to lingering sensory symptoms that adversely affect quality of life.^8,9^ Nevertheless, prior reports of oxaliplatin rechallenge or retreatment in metastatic colorectal cancer suggest that severe neuropathy occurs in a minority of patients and that re-exposure does not universally exacerbate pre-existing neuropathy.^46,47^ In this study neuropathy during prior treatment was included in the PS-IPW model to mitigate confounding, but systematic neurotoxicity grading and quality-of-life data were not available. The exploratory subgroup analysis suggested a numerical trend favoring paclitaxel-ramucirumab among patients who experienced neuropathy during first-line treatment. Therefore, the risk that oxaliplatin reintroduction may exacerbate neuropathy or impact quality of life should be carefully weighed in clinical decision-making.

## Limitations

A limitation of this study is that we were only able to include 129 patients—of which 121 were used for analysis due to trimming—partially due to the fact that the NCR exclusively acquired data of second-line treatment regimens between 2015 and 2018, but also due to the selectivity of this population. Although PS-IPW created a pseudopopulation of 241.5 patients, our results have not been sufficiently powered and even less so in the subgroup analysis, this uncertainty is reflected by the wide 95% CIs. We did perform a multivariable-adjusted Cox model as a sensitivity analysis. While the magnitude between marginal (PS-IPW) and conditional (Cox) HRs differed, this was expected due to the non-collapsibility of the HR. Importantly, the direction and statistical significance of the associations were generally consistent across approaches, providing additional reassurance regarding the observed treatment effect.

Moreover, propensity score inverse probability weighting was performed using age, sex, performance status, LDH, number of metastatic sites, primary tumor location, HER2 status, duration of first-line treatment, and neuropathy during first-line therapy. These variables were selected based on clinical relevance to treatment selection and their effect on OS. Despite these adjustments, residual confounding is likely, as factors such as cumulative toxicity, disease tempo, or tumor burden are difficult to fully capture in retrospective data. Consequently, causal interpretation of the observed associations should be made with caution.

Finally, detailed variables of important clinical data were lacking. For example, (radiological) progression data after second-line were largely unavailable, such as PFS, time to treatment failure, and objective response rate—only start of third-line treatment would approach these outcomes. Also, second-line treatment intensity, dose modifications, and reasons for treatment discontinuation were largely unavailable. While the number of delivered cycles was reported, the lack of comprehensive toxicity, discontinuation information, and data on progression on second-line limits conclusions regarding treatment tolerability, particularly with respect to oxaliplatin-associated neurotoxicity.

These results alone cannot unequivocally inform treatment selection, due to the nonsignificant results and the wide confidence intervals, as well as the likelihood of residual and unmeasured confounding despite the PS-IPW. Nevertheless, the application of this target trial emulation framework together with PS-IPW informed by the DAG provides the closest feasible approximation to a randomized controlled trial given the available retrospective data.

## Conclusion

In conclusion, oxaliplatin reintroduction was associated with similar OS compared with paclitaxel-ramucirumab. Due to the limited power of our study, it is not possible to definitively distinguish between a moderate survival difference or no effect on survival. Subgroup analyses were particularly limited by small sample sizes and few events, rendering them strictly exploratory. Nevertheless, in the absence of evidence for large survival differences, treatment selection between these regimens may reasonably be guided by factors such as toxicity, prior response, and patient preference.

## Supplemental Material

sj-docx-1-tam-10.1177_17588359261442616 – Supplemental material for Second-line oxaliplatin reintroduction or paclitaxel-ramucirumab in metastatic gastroesophageal cancer: a population-based study Supplemental material, sj-docx-1-tam-10.1177_17588359261442616 for Second-line oxaliplatin reintroduction or paclitaxel-ramucirumab in metastatic gastroesophageal cancer: a population-based study by Sebastiaan N. Siegerink, Steven C. Kuijper, Tom van den Ende, Rob H. A. Verhoeven, Sjoerd G. Elias, Nadia Haj Mohammad, Marije Slingerland, Sarah Derks and Hanneke W. M. van Laarhoven in Therapeutic Advances in Medical Oncology

sj-docx-2-tam-10.1177_17588359261442616 – Supplemental material for Second-line oxaliplatin reintroduction or paclitaxel-ramucirumab in metastatic gastroesophageal cancer: a population-based study Supplemental material, sj-docx-2-tam-10.1177_17588359261442616 for Second-line oxaliplatin reintroduction or paclitaxel-ramucirumab in metastatic gastroesophageal cancer: a population-based study by Sebastiaan N. Siegerink, Steven C. Kuijper, Tom van den Ende, Rob H. A. Verhoeven, Sjoerd G. Elias, Nadia Haj Mohammad, Marije Slingerland, Sarah Derks and Hanneke W. M. van Laarhoven in Therapeutic Advances in Medical Oncology

## Declarations

**Ethics approval and consent to participate:** This observational study does not require approval from an Ethical Committee in the Netherlands according to the Central Committee on Research Involving Human Subjects (CCMO). Use of the anonymous data for our study was approved by the Privacy Review Board of the Netherlands Cancer Registry, following the principles of the Code of Conduct for Health Research (Gedragscode Gezondheidsonderzoek—Coreon). Consent to participate was not applicable for this research.

**Consent for publication:** Not applicable.

**Availability of data and materials:** The data underlying this article will be shared on reasonable request to the corresponding author.
