# Supplementary material for: Second-line oxaliplatin reintroduction or paclitaxel-ramucirumab in metastatic gastroesophageal cancer: a population-based study
Source: Ther Adv Med Oncol. 2026 Jun 3;18:17588359261442616. doi: 10.1177/17588359261442616 (PMC13237275; doi:10.1177/17588359261442616)
Supplement: sj-docx-1-tam-10.1177_17588359261442616 – Supplemental material for Second-line oxaliplatin reintroduction or paclitaxel-ramucirumab in metastatic gastroesophageal cancer: a population-based study [file sj-docx-1-tam-10.1177_17588359261442616.docx]

**
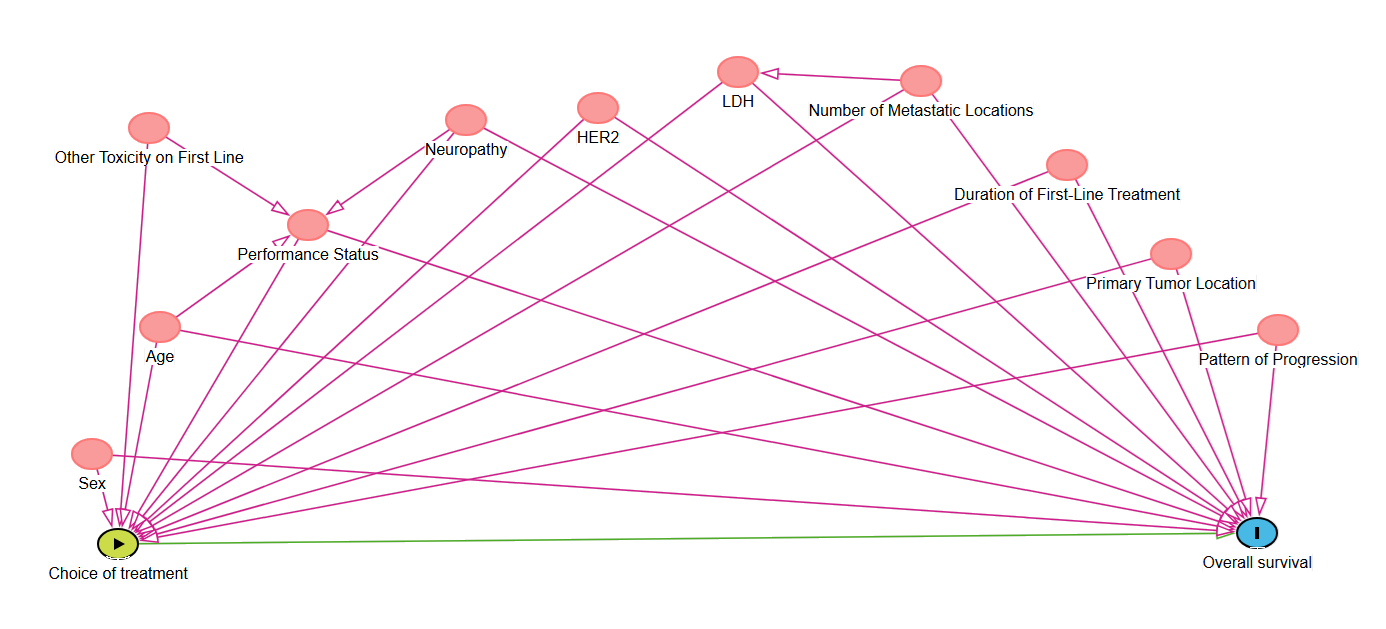
Supplementary Figures**

**Supplementary Figure 1.** Directed Acyclic Graph (DAG) illustrating possible confounders for the treatment effect. Above the horizontal line are possible confounders accounted for in the PS-IPW model.


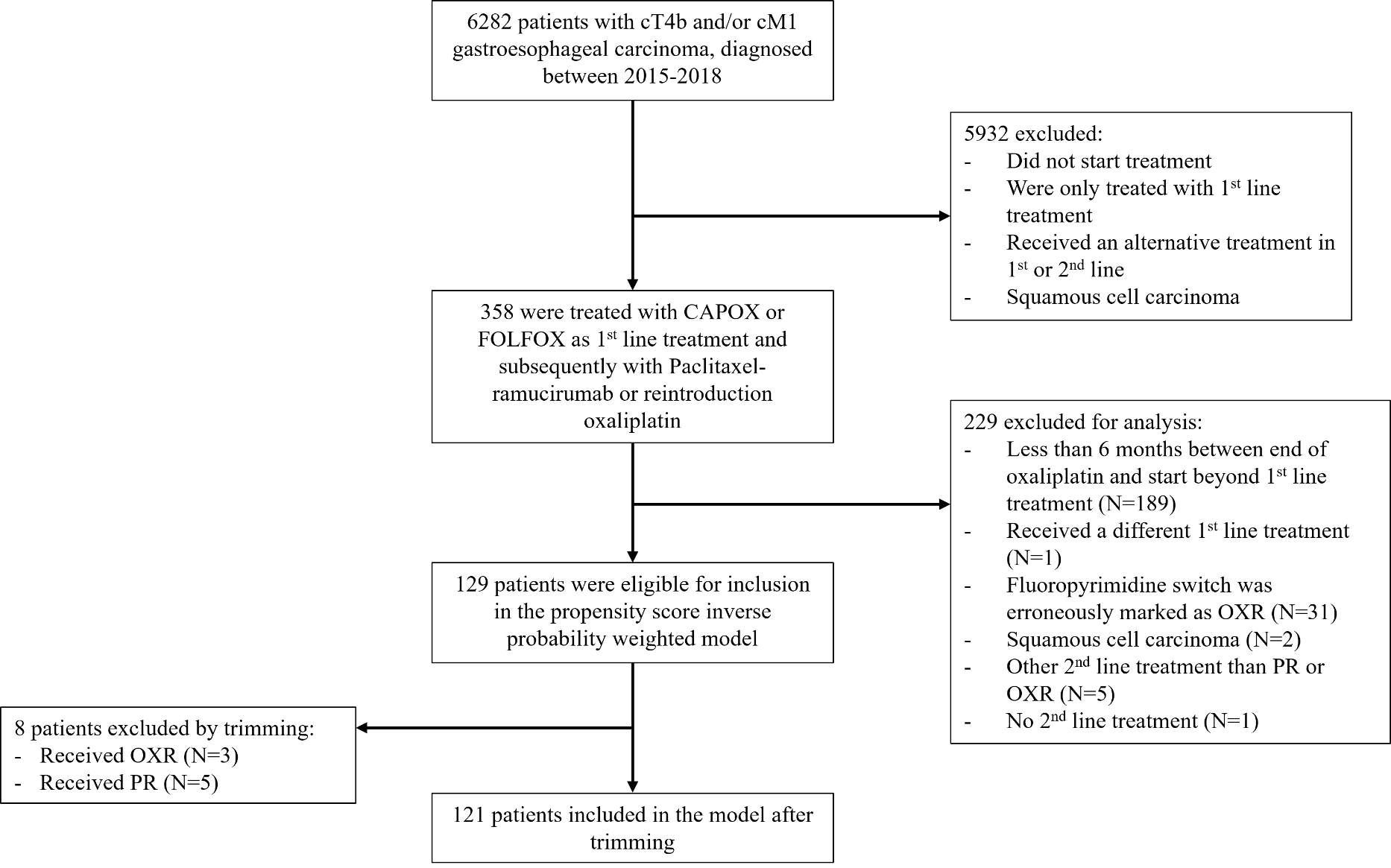


**Supplementary Figure 2.** Flow diagram of patient selection from the Netherlands Cancer Registry and inclusion in the PS-IPW cohort.

|  | **Reintroduction oxaliplatin (N = 50)** | | **Paclitaxel-ramucirumab (N=74)** | |
| --- | --- | --- | --- | --- |
| Number of Treatment Lines Beyond Second Line | N (%) | | N (%) | |
| 0 | Total | 28 (56) | Total | 58 (78.3) |
| 1 | Paclitaxel + Ramucirumab  Fluoropyrimidine + oxaliplatin  Paclitaxel monotherapy  Fluoropyrimidine + irinotecan | 15 (30)  2 (4)  2 (4)  1 (2) | Fluoropyrimidine + oxaliplatin  Irinotecan  Trifluridine/​tipiracil  Pembrolizumab  Paclitaxel + regorafenib  Tremelimumab  Fluoropyrimidine + irinotecan | 3 (4.1)  2 (2.7)  2 (2.7)  1 (1.4)  1 (1.4)  1 (1.4)  1 (1.4) |
|  | Total | 22 (44) | Total | 11 (14.9) |
| 2 | Fluoropyrimidine + oxaliplatin –  Paclitaxel + ramucirumab | 1 (2) | Fluoropyrimidine + oxaliplatin – Irinotecan  Fluoropyrimidine + oxaliplatin – Trifluridine/​tipiracil  2x Paclitaxel + ramucirumab | 1 (1.4)  1 (1.4)  1 (1.4) |
|  | Total | 1 (2) | Total | 3 (4.1) |
| 3 | 2x Fluoropyrimidine + oxaliplatin –  Paclitaxel + ramucirumab | 1 (2) | 2x Fluoropyrimidine + oxaliplatin – Irinotecan  Trifluridine/​tipiracil – Irinotecan –  Tegafur/​gimeracil/​oteracil + Dacomitinib | 1 (1.4)  1 (1.4) |
|  | Total | 1 (2) | Total | 2 (2.7) |

**Supplementary Table 1.** Overview of treatment regimens after reintroduction of oxaliplatin or after paclitaxel-ramucirumab. Not adjusted by PS-IPW due to the heterogeneity of treatment options.
